# Lipoprotein markers associated with disability from multiple sclerosis

**DOI:** 10.1038/s41598-018-35232-7

**Published:** 2018-11-19

**Authors:** A. R. Gafson, T. Thorne, C. I. J. McKechnie, B. Jimenez, R. Nicholas, P. M. Matthews

**Affiliations:** 1 Division of Brain Sciences, Department of Medicine, Imperial College, London, UK; 2 St Edmund Hall, Oxford University, Oxford, UK; 3 Clinical Phenotyping Centre, Division of Computational Systems Medicine. Department of Surgery & Cancer, Imperial College, London, UK; 4 Centre for Neurotechnology and UK Dementia Research Institute, Imperial College, London, UK

## Abstract

Altered lipid metabolism is a feature of chronic inflammatory disorders. Increased plasma lipids and lipoproteins have been associated with multiple sclerosis (MS) disease activity. Our objective was to characterise the specific lipids and associated plasma lipoproteins increased in MS and to test for an association with disability. Plasma samples were collected from 27 RRMS patients (median EDSS, 1.5, range 1–7) and 31 healthy controls. Concentrations of lipids within lipoprotein sub-classes were determined from NMR spectra. Plasma cytokines were measured using the MesoScale Discovery V-PLEX kit. Associations were tested using multivariate linear regression. Differences between the patient and volunteer groups were found for lipids within VLDL and HDL lipoprotein sub-fractions (p < 0.05). Multivariate regression demonstrated a high correlation between lipids within VLDL sub-classes and the Expanded Disability Status Scale (EDSS) (p < 0.05). An optimal model for EDSS included free cholesterol carried by VLDL-2, gender and age (R^2^ = 0.38, p < 0.05). Free cholesterol carried by VLDL-2 was highly correlated with plasma cytokines CCL-17 and IL-7 (R^2^ = 0.78, p < 0.0001). These results highlight relationships between disability, inflammatory responses and systemic lipid metabolism in RRMS. Altered lipid metabolism with systemic inflammation may contribute to immune activation.

## Introduction

Multiple Sclerosis (MS) is a chronic neuroinflammatory disorder and one of the most common non-traumatic causes of acquired disability in young adults^1^. Monitoring progression currently relies on insensitive clinical measures and serial brain imaging, as there are no qualified blood biomarkers of disease progression^2^. Identifying such markers could both enable more accurate patient stratification and monitoring of therapeutic interventions.

The association between inflammation and alterations in lipid metabolism is well characterised. The pro-inflammatory state, characterised by an immune-cell mediated release of cytokines, can cause a rise in triglyceride rich serum lipoproteins secondary to increases in production and reduced hepatic clearance^3,4^. This furthers inflammatory cascades through the production of yet more pro-inflammatory cytokines by macrophages, for example^5^. This pattern, as well as an increase in vascular comorbidity are features of a number of chronic systemic autoimmune diseases such as rheumatoid arthritis, systemic lupus erythematosus and psoriasis^3,6,7^.

Previous studies have suggested an association between dyslipidaemia, dyslipoproteinaemia and greater MS disease activity (new MRI lesions^8,9^ or worsening EDSS^8,10,11^). Furthermore, large metabolomics studies have demonstrated that fatty acids, triglycerides, cholesterols and phospholipids can accurately distinguish MS patients from healthy controls as well as those with greater clinical disability or at different stages of disease^12–14^. Lipid dysregulation and dyslipoproteinaemia may occur as a secondary by-product of myelin destruction in the central nervous system^12^. Alternatively, they may play a role in immune dysfunction through regulation of cytokine production or increased transportation on lipid rafts^15^. Recently, indirect evidence consistent with a causal role for dyslipidaemia in disease progression was provided by results from MS-STAT, a Phase IIa study showing that high dose simvastatin reduced rates of brain atrophy and disability progression in patients with secondary progressive MS^16^. Similar findings were not observed in a trial combining atorvastatin with interferon beta suggesting that this interaction may be complex^17^.

Lipids and lipoproteins can be measured using a number of techniques including ultracentrifugation, high performance liquid chromatography and nuclear magnetic resonance (NMR) spectroscopy^18^. A novel NMR method has recently been developed that can measure lipid concentrations (cholesterol, free cholesterol, triglyceride, phospholipids and apolipoproteins) within plasma lipoprotein sub-fractions (lipoproteins subdivided based on density and size). This method has the advantages of being amenable to high throughput use, is highly reproducible and can provide simultaneous class-specific information on both lipoproteins and their constituent lipids, both of which have been associated with MS disease activity^19^. It promises increased sensitivity to changes in circulating lipids related to systemic inflammatory states. Here we tested for an association between MS and lipid concentrations within plasma lipoprotein sub-fractions in relapsing-remitting MS patients (RRMS) relative to age- and sex- matched healthy volunteers. We also tested for association between these lipid concentrations and plasma cytokines, as markers of both systemic inflammatory responses and disability.

## Methods

Our research study was reviewed and approved by the NREC Committee of London Camden and Islington (NREC 14/LO/1896). All methods were performed in accordance with the relevant guidelines and regulations. All patients provided full, informed written consent to participate in the study.

### Study Design

Our cohort included 27 patients with RRMS (RRMS; median EDSS, 1.5, range 1–7) diagnosed by McDonald criteria^20^, who were recruited from the Imperial College Healthcare NHS Trust prior to commencing a new disease modifying treatment and who consented for participation in the study (Table 1). Patients recruited were aged between 18–65 and treatment-free (disease modifying treatments and steroids) for at least 3 months as well as relapse-free for at least a month. Any subjects on statin therapies were excluded from the analysis. Three patients experienced regular migraines, 2 had asthma, 2 psoriasis and 2 had autoimmune thyroid disorders. 31 age- and gender- matched healthy controls were recruited by local advertising or commercially sourced. The Expanded Disability Status Score (EDSS) was used to assess disability in the patients and conducted by a single, trained physician (AG).

**Table 1:** Patient and healthy volunteer demographic data.

	MS Patients		Controls
Gender	Men (n = 12)	Women (n = 15)	17 Men 14 Women
Mean Age (years)	41.0 ± 13.4	41.9 ± 12.3	40.3 ± 8.6
Average disease duration from diagnosis (years)	4 ± 4	5 ± 4	*N/A*
Average disease duration from first symptom (years)	9 ± 7	9 ± 7	*N/A*
EDSS (median, range)	1.5 (1–7.0)	3.0 (1–6.5)	*N/A*
Treatment Naïve patients	7	7	*N/A*
Current Smoker	2	1	0

Values quoted as mean ± standard deviation if not indicated otherwise.

### Sample Collection

*Non*-fasting venous blood samples were collected at study visits in EDTA tubes and centrifuged at 1400 × g for 10 minutes within 3 hours of sample collection. Plasma then was separated immediately into aliquots of 1 ml and stored at −80 °C.

### NMR Spectroscopy

Plasma samples were centrifuged for 5 minutes at 4 °C at 13,000 rpm to remove solid particles in suspension. Samples were prepared as previously described for NMR analysis^21^. A Bruker 600 Avance III spectrometer was used to acquire a 1D NMR general profile, spin-echo and 2D J-res spectra^21^. Spectra were processed, phased and automatically baseline corrected using TopSpin software (v3.2, Bruker, BioSpin, Germany). The signal from the anomeric proton of the glucose at 5.23 ppm was used to calibrate the plasma spectra.

### Lipoprotein and cytokine analyses

Plasma lipoprotein quantification was performed with the Bruker B.I.-LISA (Bruker IVDr Lipoprotein Subclass Analysis) platform using the –CH_3_ and CH_2_ resonances in the ^1^H-general profile NMR spectrum, which appear at 0.85 and 1.20 ppm, respectively. These broad resonances were bucketed and fitted against a Partial Least Square (PLS2) regression model. The model has been validated against direct assays after plasma ultracentrifugation^19^. For each sample, the method estimates total plasma triglyceride, cholesterol, free cholesterol, phospholipid and apolipoprotein A1, A2 and B. It also estimates concentrations of these lipids (where calculable) within the main lipoprotein classes (VLDL, IDL, LDL, HDL), subdivided according to increasing density and decreasing size (VLDL-1 - VLDL-6, LDL-1 - LDL-6, HDL-1 - HDL-4). 105 lipoprotein sub-fractions were analysed in each sample using this method and a complete list of these are identified in Supplementary Table 1. A test-retest comparison of sub-fractions measured in a single healthy control from samples taken on 5 consecutive days showed that mean lipoprotein sub-fraction concentrations varied by <5%.

Plasma cytokine analysis was performed using the Meso Scale Discovery (MSD) V-PLEX kit (measuring concentrations of 40 cytokines) run as recommended by the manufacturer (Meso Scale Discovery, Maryland, USA). Results were read with a MSD Sector Imager 6000 and cytokine concentrations determined with Softmax Pro Version 2.5 software using curve fit models.

### Statistical Analyses

Descriptive statistics were used to summarise MS patient and healthy control demographics. For lipoprotein analyses, contrasts between patients and controls were performed using ANOVA (F test). If the ANOVA showed a statistically significant difference (p < 0.05), a *post hoc* analysis was performed using Holm-Bonferroni to correct for multiple comparisons. The desired level of significance was set at p < 0.05 after correction.

**Figure 1:**
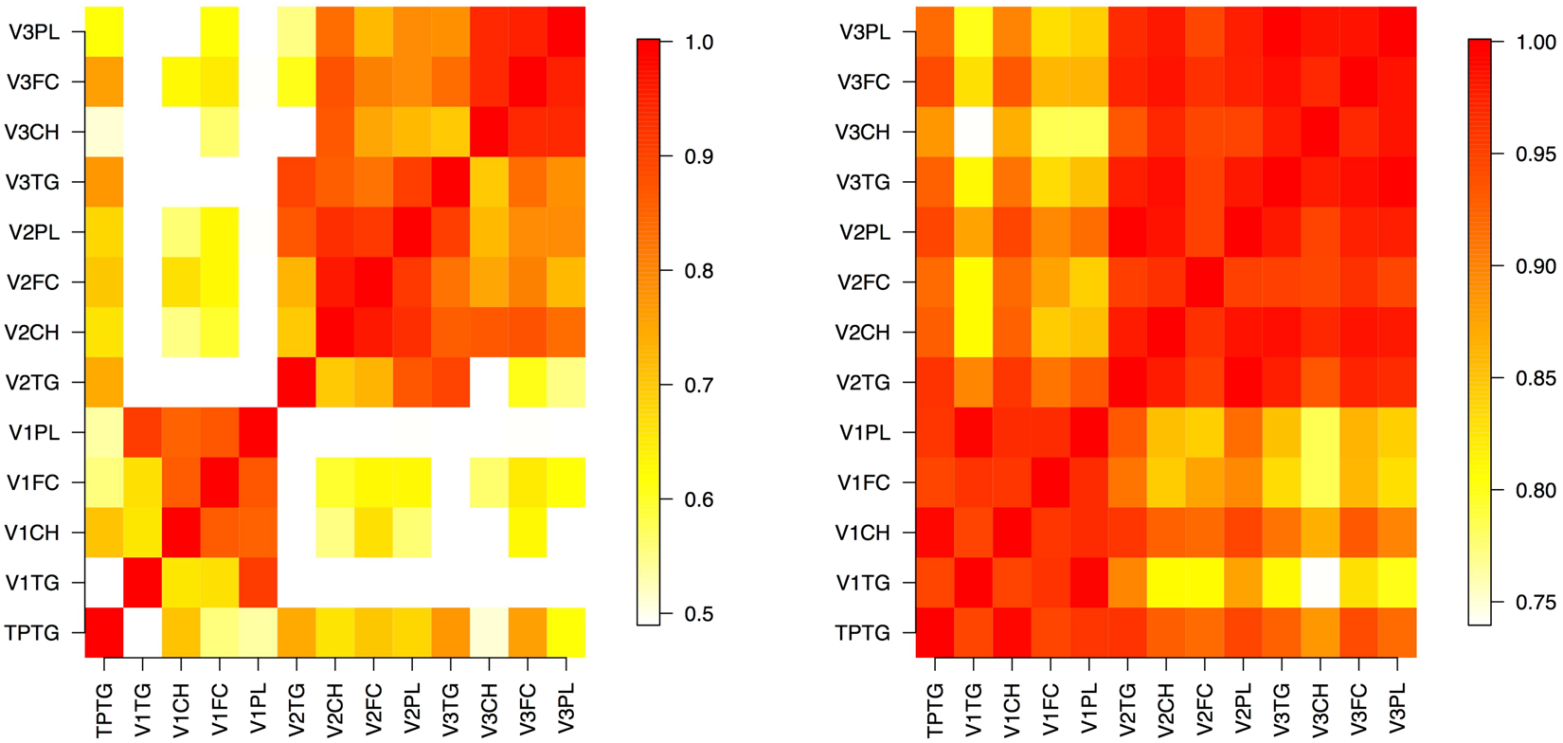
(**a,b**) Matrices illustrating pairwise correlations between VLDL sub-fraction concentrations for healthy volunteers (**a**) and MS patients (**b**). Abbreviations: TPTG = total plasma triglycerides, V1TG = VLDL-1; triglycerides, V1CH = VLDL-1; cholesterol, V1FC = VLDL-1; free cholesterol, V1PL = VLDL-1; phospholipids, V2TG = VLDL-2; triglycerides, V2CH = VLDL-2; cholesterol, V2FC = VLDL-2; free cholesterol, V2PL = VLDL-2; phospholipids, V3TG = VLDL-3; triglycerides, V3CH = VLDL-3; cholesterol, V3FC = VLDL-3; free cholesterol, V3PL = VLDL-3, phospholipids. Bar represents r-value for pairwise correlation.

Lipids within Lipoprotein sub-fractions that had different concentrations between patients and controls with a fold change >1.30 were taken forward for further analyses. Pearson’s correlation coefficients between each pair of VLDL sub-fractions were calculated and used to create the matrix of pairwise correlations (Fig. 1a,b).

Correlations between EDSS and lipids and MS patient characteristics were analysed using multivariate linear regression models. Due to the high collinearity between the lipids within lipoprotein sub-fractions, which would have led to variance inflation in a model^22^, each lipid sub-fraction was analysed separately using the regression model. We included well recognised risk factors of age, gender, disease duration and body mass index (BMI)^1^. EDSS was defined as the dependent variable, with the patient characteristics and lipid measures as predictive variables:

$${\rm{EDSS}}={{\rm{\alpha }}}_{1}{\rm{Lipid}}\,({\rm{within}}\,{\rm{lipoprotein}})+{{\rm{\alpha }}}_{2}{\rm{Age}}+{{\rm{\alpha }}}_{3}{\rm{Gender}}+{{\rm{\alpha }}}_{4}{\rm{Disease}}\,{\rm{Duration}}+{{\rm{\alpha }}}_{5}{\rm{BMI}}+{{\rm{\alpha }}}_{6}.$$

where coefficients α_1,…,_α_5_ determine the contributions of each of the predictor variables to EDSS and α_6_ expresses the residual error. We used analysis of residuals to check the required assumptions of normally distributed errors with constant variance. Regression models were corrected for multiple comparisons using Holm-Bonferroni method.

These multivariate regression models then were evaluated from their R^2^ values, residual standard errors and the overall p-values. The level of significance was set at p < 0.05. The relative significance of contributions of individual predictive variables was assessed by averaging sequential sums of squares obtained from all possible orderings of the predictors using the LMG method^23^. Statistics were evaluated using ‘relaimpo’ package in R^24^.

We used the Akaike Information Criterion (AIC) to determine the most parsimonious model with the predictor variables available^25^. These included age, disease duration, sex, BMI and those lipids that were both raised in patients compared to controls *and* correlated with EDSS in the prior multivariate regression analyses. AIC allows us to perform model selection to derive a preferred model based on a trade-off between goodness of fit and model complexity. These statistics were analysed using the ‘mass’ package in R^26^. In order to confirm stability of the resulting model was unstable, we performed a leave-one-out cross validation removing one patient in turn and recording the p-values for each derived model.

To explore associations between cytokines and lipids, we created regression models using the lipid included in the most parsimonious model derived from AIC (free cholesterol carried by VLDL-2) and input this into a generalised linear model via penalised maximum likelihood. To improve the interpretability and accuracy given the large number cytokine variables, we used a lasso regression based cross–validation^27^. Analyses were performed using the glmnet package in R^28^.

## Results

Patient demographics and clinical information are provided in Table 1. 13 patients had been on previous disease modifying treatment, 14 patients were treatment-naïve. One patient was excluded from the study due to concurrent statin treatment.

### Lipids within lipoproteins are increased in MS patients compared to healthy controls

A global contrast was performed between measures for the MS patients and healthy volunteer groups. We found 23 lipids within VLDL and HDL sub-fractions that were increased in the patient group relative to the healthy volunteers (Table 2). 13/23 of these lipid concentrations had changes of >1.30-fold in patients relative to the volunteers (range, 1.31–2.00-fold) (Table 2). Concentrations of total plasma cholesterol and total plasma triglycerides are defined in Supplementary Table 2.

**Table 2:** Lipids within VLDL and HDL lipid sub-fractions that were significantly increased in MS patients relative to healthy volunteers. Concentrations are quoted in mg/dL ± standard deviation.

Lipid Sub-Fraction	Concentration (mg/dL)		Relative Change (patients/controls)	F Test (corrected)
	*Patient*	*Control*		
VLDL-2				
Free Cholesterol	1.27 (±1.32)	0.97 (±0.81)	1.31	p = 0.03
Triglycerides	13.66 (±9.99)	9.72 (±5.96)	1.40	p = 0.03
Phospholipids	3.21 (±2.46)	2.59 (±1.45)	1.24	p = 0.03
Cholesterol	3.02 (±2.96)	2.04 (±1.47)	1.48	p = 0.003
VLDL-3				
Free Cholesterol	1.49 (±1.50)	1.26 (±0.90)	1.18	p = 0.03
Triglycerides	11.40 (±8.78)	9.00 (±5.02)	1.27	p = 0.03
Phospholipids	3.39 (±2.80)	3.08 (±1.64)	1.10	p = 0.03
Cholesterol	3.67 (±3.60)	3.08 (±1.86)	1.20	p = 0.007
VLDL- 4				
Free Cholesterol	2.21 (±1.97)	1.98 (±1.13)	1.11	p = 0.03
Triglycerides	8.04 (±5.23)	7.88 (±3.08)	1.02	p = 0.03
Cholesterol	4.82 (±4.17)	4.62 (±2.34)	1.04	p = 0.02
HDL- 1				
Apolipoprotein-A1	34.4 (±19.1)	20.1 (±9.3)	1.7	p = 0.002
Apolipoprotein-A2	3.70 (±1.92)	1.85 (±1.04)	2.00	p = 0.01
Phospholipids	27.70 (±13.00)	17.00 (±7.11)	1.62	p = 0.02
Cholesterol	22.66 (±10.44)	14.56 (±5.80)	1.56	p = 0.02
Free Cholesterol	7.30 (±2.56)	4.18 (±1.55)	1.74	p = 0.03
HDL- 2				
Apolipoprotein-A1	21.40 (±6.81)	15.69 (±3.88)	1.36	p = 0.03
Apolipoprotein-A2	4.17 (±1.59)	2.69 (±0.91)	1.55	p = 0.03
Phospholipids	15.93 (±5.67)	10.78 (±3.22)	1.48	p = 0.03
Cholesterol	10.73 (±3.59)	7.28 (±2.19)	1.47	p = 0.03
Triglycerides	1.95 (±1.00)	1.83 (±0.62)	1.06	p = 0.04
HDL- 3				
Apolipoprotein-A2	8.09 (±2.44)	5.78 (±1.42)	1.40	p = 0.03
Triglycerides	2.22 (±1.04)	2.07 (±0.60)	1.07	p = 0.03

### Lipids within VLDL sub-fractions are correlated with disability in people with MS

We then tested whether lipid concentrations within lipoprotein sub-fractions elevated in our cohort were correlated with MS disease disability. To do this, we used a multivariate regression approach accounting for variance from other potential major determinants (see Methods). We found that concentrations of two of the lipids in VLDL-2 sub-fractions (cholesterol and free cholesterol) but none of the lipids in any HDL sub-fractions were correlated with EDSS (Table 3). Strong pairwise correlations were also observed between the relative concentrations of lipids within VLDL lipid sub-fractions in the patient and healthy volunteer groups (r >0.5 for all) (Fig. 1a,b).

**Table 3:** Statistical measures of fit for each lipid within VLDL and HDL sub-fractions with corresponding corrected p-value, residual standard errors for the regression model and corrected p-value for the lipid predictor coefficient. NS = not significant.

Lipid Sub-Fraction	Model R^2^	Model P-value	Lipid P - value	Residual Error
VLDL-2				
Free Cholesterol	0.40	p = 0.0013	p = 0.026	1.78
Triglycerides	0.39	p = 0.0026	p = 0.052	1.80
Cholesterol	0.39	p = 0.0026	p = 0.030	1.79
HDL-1				
Apolipoprotein-A1	0.28	NS	NS	2.08
Apolipoprotein-A2	0.27	NS	NS	2.09
Phospholipids	0.28	NS	NS	2.08
Cholesterol	0.29	NS	NS	2.06
Free Cholesterol	0.29	NS	NS	2.06
HDL- 2				
Apolipoprotein-A1	0.27	NS	NS	2.10
Apolipoprotein-A2	0.28	NS	NS	2.08
Phospholipids	0.28	NS	NS	2.08
Cholesterol	0.27	NS	NS	2.09
HDL- 3				
Apolipoprotein-A2	0.30	NS	NS	2.05

**Figure 2:**
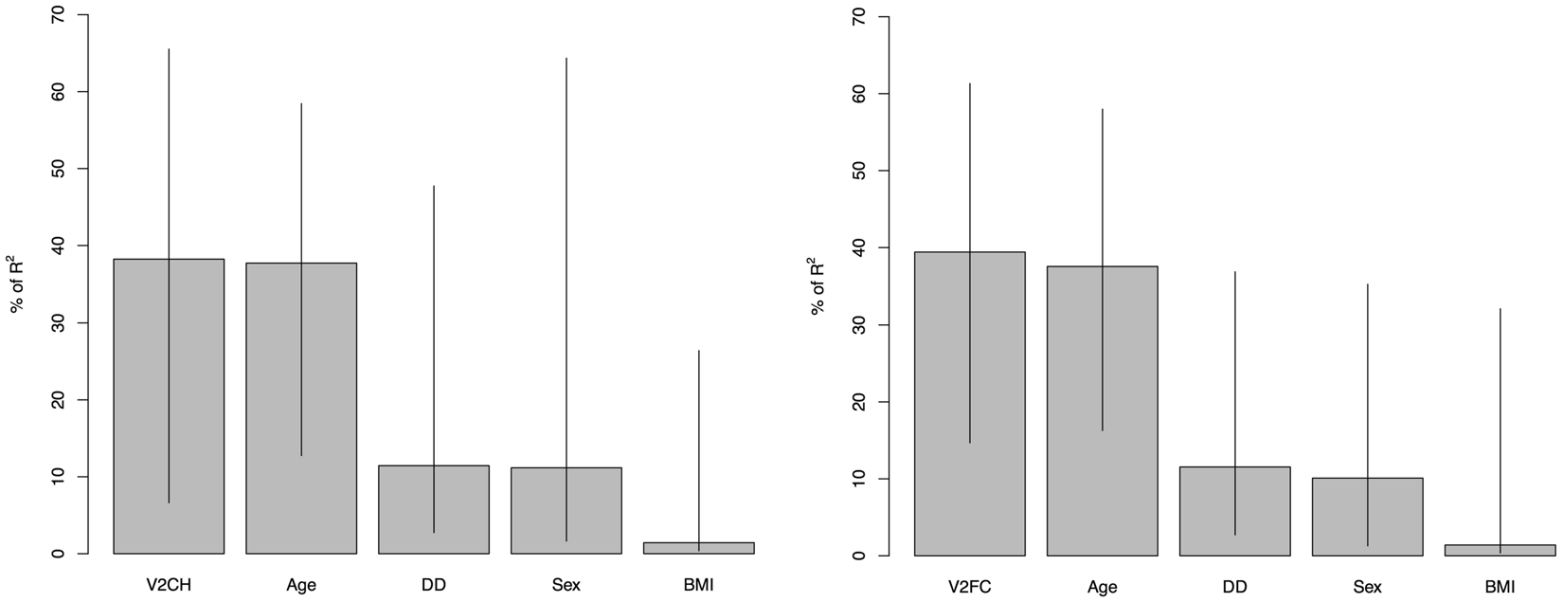
(**a,b**) Relative weighting of variables to multivariate regression models for VLDL-2 (free cholesterol and cholesterol). Values expressed as percentage of R^2^ with 95% confidence intervals. Abbreviations: DD = Disease Duration; V2CH = VLDL-2; cholesterol, V2FC = VLDL-2; free cholesterol.

We assessed the relative contibution of lipid concentrations in VLDL-2 for explaining EDSS using a residual sum of squares approach. Total and free cholesterol concentrations in the VLDL-2 sub-fractions accounted for the greatest proportions of variance (0.38 and 0.39, respectively) (Fig. 2a,b).

We selected an optimal model based on Akaike Information Criterion (AIC) after incorporating total cholesterol and free cholesterol within VLDL-2 and clinical characteristics. The best quality model excluded BMI, disease duration and cholesterol, leaving free cholesterol in VLDL-2, age and gender as the most informative explanatory variables. This multivariate regression model described a moderate correlation for EDSS (R^2^ = 0.37, p = 0.0001). The contribution of free cholesterol in VLDL-2 to this model was independently significant (p = 0.003). We tested stability of the model using leave-one-out cross validation (LOOCV). For each run of LOOCV, the model remained significant (Supplementary Table 3).

### Increased plasma cytokine concentrations are associated with raised free cholesterol concentrations in VLDL sub-fractions in MS patients

We then tested whether increased plasma cytokines, as measures of systemic inflammatory state, were related to concentrations of free cholesterol within the VLDL-2 lipoprotein sub-fraction. Free cholesterol concentrations within VLDL-2 were used in a general linear model to test for associations with plasma cytokine levels. Strong correlations between cholesterol in VLDL-2 and both CCL-17 and IL-7 concentrations were found (R^2^ = 0.78, p < 0.0001).

## Discussion

Dyslipidaemia has previously been associated with MS disease activity. Here we extended these observations by characterising associations of specific plasma lipoprotein sub-fractions and the concentration of lipids carried within them with disease and disability level in people with RRMS. Using a novel NMR analytical methodology, we showed that specific lipid concentrations within VLDL and HDL lipoprotein sub-fractions are increased in MS patients relative to healthy subjects. We additionally observed correlations between individual lipids carried by the VLDL-2 sub-fraction (cholesterol, free cholesterol and triglycerides) and measures of clinical disability (EDSS) in the MS patients. Separate analyses based on concentrations of these lipids within VLDL-2 showed that total and free cholesterol were the strongest explanatory variables for EDSS. The correlation of free cholesterol within VLDL-2 and plasma concentrations of the CCL-17 and IL-7 cytokines both highlights that systemic inflammation alters lipid metabolism and suggests that altered lipid metabolism may enhance immune responses.

This study took an unbiased approach by initially performing a global lipoprotein analysis using a recently developed NMR spectroscopy method that allows the simultaneous quantification of a large number of lipid concentrations within lipoprotein sub-fractions^19^. This method is unique from traditional lipoprotein quantification as it takes into account the concentrations of water-insoluble lipids within the supermolecular assemblies. Lipids within VLDL and HDL sub-fractions were consistently elevated in the patient cohort compared with healthy volunteers. Relative concentrations of a subset of these lipids within VLDL-2 sub-fractions only were correlated with disease disability. Whilst age and gender have previously been associated with a poorer prognosis in MS^29^, VLDL-2 (free cholesterol and cholesterol) sub-fractions independently explained as much of the variance in disability as age.

The relationship is not new. A number of previous studies have reported that plasma lipids such as cholesterol and their carrier lipoproteins are raised in MS and that increases are correlated with disease disability^9,10,30,31^. A recent paper corroborates our findings of both raised triglyceride-rich VLDL and HDL in RRMS patients compared to controls^32^. The protective association of HDL with MS severity is consistent with our finding that lipids within HDL specifically were not associated with worsening MS disability.

In this study, we identified novel associations between specific lipids within VLDL sub-fractions and MS disability. Increases in VLDL levels^33^ also have been reported during other inflammatory disorders such as rheumatoid arthritis and systemic lupus erythematosus but have not been identified in MS^3^. The mechanisms responsible for the VLDL increases in MS reported here were not investigated directly. However, cytokines increase production and reduce clearance of serum triglycerides and VLDL^4^. Triglyceride-rich VLDL (as well as VLDL remnants resulting from lipolysis)^34,35^ is taken up by monocytes, where it can stimulate pro-inflammatory cytokine production via activation of the MAPK pathway, for example^5,36^. A possible relationship with disability may arise from consequently greater numbers of activated monocytes recruited to acute plaques and greater demyelination^37^, as well as exacerbation of blood-brain barrier disruption^38^.

Here we observed a strong correlation between free cholesterol within VLDL-2 and both CCL-17 and IL-7 highlighting a potential interaction between inflammation and dyslipidaemia. CCL-17 has been shown to play a role in T cell maturation. A common polymorphism in the IL-7 receptor gene is a known genetic risk factor for MS^39^. Statins are reported to lower both VLDL^40,41^ and various cytokines including IL-7^42^.

The main limitations of this pilot study were methodological. Our samples were taken non-fasting and our healthy control population was not matched for diet with our MS population. However, this would have altered a urine metabolomic profile more than a plasma metabolomic profile and we included BMI as a potential confounder in the regression model. The cross-sectional nature of the study, the small sample size and the lack of a validation cohort were further limitations however the use of leave-one out cross validation removed the potential effect of an outlier. While commonly used in the general population, only one patient in our study group was on lipid-lowering medication, and this patient was eliminated from the analyses.

In conclusion, we have provided evidence that specific lipid concentrations within VLDL sub-fractions are correlated both with disability in RRMS patients and with pro-inflammatory plasma cytokine levels. These findings highlight relationships between systemic metabolism (particularly involving the liver where VLDLs are synthesised), inflammation and MS^43^. They suggest that clinical benefits of lipid lowering drugs with inflammatory diseases may be realised by decreasing plasma lipid concentrations and, through this, by reducing associated monocyte activation. Prospective studies exploring how concentrations of lipids within VLDL sub-classes evolve through the disease course will help to determine the prognostic value of these measures in predicting disability progression.

## Electronic supplementary material

Supplementary Tables 1, 2 and 3

## Data Availability

The datasets generated during and/or analysed during the current study are available from the corresponding author on reasonable request.

## References

[1] Compston A, Coles A (2002). Multiple sclerosis. Lancet.

[2] Gafson Arie, Craner Matt J, Matthews Paul M (2016). Personalised medicine for multiple sclerosis care. Multiple Sclerosis Journal.

[3] Feingold, K. R. & Grunfeld, C. In *Endotext* (eds L. J. De Groot *et al*.) (2000).

[4] Khovidhunkit W (2004). Effects of infection and inflammation on lipid and lipoprotein metabolism: mechanisms and consequences to the host. J Lipid Res.

[5] Saraswathi V, Hasty AH (2006). The role of lipolysis in mediating the proinflammatory effects of very low density lipoproteins in mouse peritoneal macrophages. J Lipid Res.

[6] Tettey P, Simpson S, Taylor BV, van der Mei IA (2014). Vascular comorbidities in the onset and progression of multiple sclerosis. Journal of the neurological sciences.

[7] Marrie RA (2010). Vascular comorbidity is associated with more rapid disability progression in multiple sclerosis. Neurology.

[8] Weinstock-Guttman B (2011). Serum lipid profiles are associated with disability and MRI outcomes in multiple sclerosis. Journal of neuroinflammation.

[9] Browne RW (2014). Apolipoproteins are associated with new MRI lesions and deep grey matter atrophy in clinically isolated syndromes. Journal of neurology, neurosurgery, and psychiatry.

[10] Tettey P (2014). An adverse lipid profile is associated with disability and progression in disability, in people with MS. Multiple sclerosis.

[11] Mandoj C (2015). Anti-annexin antibodies, cholesterol levels and disability in multiple sclerosis. Neuroscience letters.

[12] Villoslada P (2017). Metabolomic signatures associated with disease severity in multiple sclerosis. Neurol Neuroimmunol Neuroinflamm.

[13] Dickens AM (2014). A type 2 biomarker separates relapsing-remitting from secondary progressive multiple sclerosis. Neurology.

[14] Bhargava P, Calabresi PA (2016). Metabolomics in multiple sclerosis. Multiple sclerosis.

[15] O’Neill LA, Kishton RJ, Rathmell J (2016). A guide to immunometabolism for immunologists. Nature reviews. Immunology.

[16] Chataway J (2014). Effect of high-dose simvastatin on brain atrophy and disability in secondary progressive multiple sclerosis (MS-STAT): a randomised, placebo-controlled, phase 2 trial. Lancet.

[17] Kamm CP (2014). Atorvastatin added to interferon beta for relapsing multiple sclerosis: 12-month treatment extension of the randomized multicenter SWABIMS trial. PloS one.

[18] Hafiane A, Genest J (2015). High density lipoproteins: Measurement techniques and potential biomarkers of cardiovascular risk. BBA Clin.

[19] *Bruker IVDr Lipoprotein Subclass AnalysisBruker IVDr Lipoprotein Subclass Analysis*, https://www.bruker.com/products/mr/nmr-preclinical-screening/lipoprotein-subclass-analysis.html.

[20] Polman CH (2011). Diagnostic criteria for multiple sclerosis: 2010 revisions to the McDonald criteria. Annals of neurology.

[21] Dona AC (2014). Precision high-throughput proton NMR spectroscopy of human urine, serum, and plasma for large-scale metabolic phenotyping. Anal Chem.

[22] Tu YK, Kellett M, Clerehugh V, Gilthorpe MS (2005). Problems of correlations between explanatory variables in multiple regression analyses in the dental literature. Br Dent J.

[23] Lindeman, R. H., Merenda, P. F. & Gold, R. Z. *Introduction to Bivariate and Multivariate Analysi*s. (1980).

[24] Gromping U (2006). Relative Importance for Linear Regression in R: The Package relaimpo. Journal of Statistical Software.

[25] Akaike H (1974). A new look at the statistical model identification. IEEE Transactions on Automatic Control.

[26] Ripley, W. N. V. a. B. D. Modern Applied Statistics with S. (2002).

[27] Lever J, Krzywinski M, Altman N (2016). Points of Significance: Regularization. Nature methods.

[28] Friedman J, Hastie T, Tibshirani R (2010). Regularization Paths for Generalized Linear Models via Coordinate Descent. J Stat Softw.

[29] Vukusic S, Confavreux C (2007). Natural history of multiple sclerosis: risk factors and prognostic indicators. Current opinion in neurology.

[30] Zhornitsky S, McKay KA, Metz LM, Teunissen CE, Rangachari M (2016). Cholesterol and markers of cholesterol turnover in multiple sclerosis: relationship with disease outcomes. Multiple sclerosis and related disorders.

[31] Giubilei F (2002). Blood cholesterol and MRI activity in first clinical episode suggestive of multiple sclerosis. Acta neurologica Scandinavica.

[32] Jorissen W (2017). Relapsing-remitting multiple sclerosis patients display an altered lipoprotein profile with dysfunctional HDL. Sci Rep.

[33] Hardardottir I, Grunfeld C, Feingold KR (1995). Effects of endotoxin on lipid metabolism. Biochem Soc Trans.

[34] Huff MW, Evans AJ, Sawyez CG, Wolfe BM, Nestel PJ (1991). Cholesterol accumulation in J774 macrophages induced by triglyceride-rich lipoproteins. Comparison of very low density lipoprotein from subjects with type III, IV, and V hyperlipoproteinemias. Arterioscler Thromb.

[35] Jong MC (2000). Oxidized VLDL induces less triglyceride accumulation in J774 macrophages than native VLDL due to an impaired extracellular lipolysis. Arterioscler Thromb Vasc Biol.

[36] Li Y (2005). Free cholesterol-loaded macrophages are an abundant source of tumor necrosis factor-alpha and interleukin-6: model of NF-kappaB- and map kinase-dependent inflammation in advanced atherosclerosis. J Biol Chem.

[37] Newcombe J, Li H, Cuzner ML (1994). Low density lipoprotein uptake by macrophages in multiple sclerosis plaques: implications for pathogenesis. Neuropathology and applied neurobiology.

[38] Jiang X (2012). Simvastatin blocks blood-brain barrier disruptions induced by elevated cholesterol both *in vivo* and *in vitro*. Int J Alzheimers Dis.

[39] International Multiple Sclerosis Genetics, C. *et al*. Risk alleles for multiple sclerosis identified by a genomewide study. *The New England journal of medicine* **357**, 851–862, 10.1056/NEJMoa073493 (2007). 10.1056/NEJMoa073493 17660530

[40] Jungnickel PW, Cantral KA, Maloley PA (1992). Pravastatin: a new drug for the treatment of hypercholesterolemia. Clin Pharm.

[41] Malhotra HS, Goa KL (2001). Atorvastatin: an updated review of its pharmacological properties and use in dyslipidaemia. Drugs.

[42] Thomson NC (2015). Atorvastatin in combination with inhaled beclometasone modulates inflammatory sputum mediators in smokers with asthma. Pulm Pharmacol Ther.

[43] Gibbons G.F., Wiggins D., Brown A.-M., Hebbachi A.-M. (2004). Synthesis and function of hepatic very-low-density lipoprotein. Biochemical Society Transactions.

