# Supplementary material for: Lipoprotein markers associated with disability from multiple sclerosis
Source: Sci Rep. 2018 Nov 19;8:17026. doi: 10.1038/s41598-018-35232-7 (PMC6242870; doi:10.1038/s41598-018-35232-7)
Supplement: Supplementary file 1 — Supplementary Tables 1, 2 and 3 [file 41598_2018_35232_MOESM1_ESM.docx]

|  | Triglyceride | Cholesterol | Free Cholesterol | Phospholipids | Apo-A1 | Apo-A2 | Apo-B |
| --- | --- | --- | --- | --- | --- | --- | --- |
| Plasma | **√** | **√** | **√** | **-** | **√** | **√** | **√** |
| All VLDL | **√** | **√** | **√** | **√** | **-** | **-** | **√** |
| VLDL-1 | **√** | **√** | **√** | **√** | **-** | **-** | **-** |
| VLDL-2 | **√** | **√** | **√** | **√** | **-** | **-** | **-** |
| VLDL-3 | **√** | **√** | **√** | **√** | **-** | **-** | **-** |
| VLDL-4 | **√** | **√** | **√** | **√** | **-** | **-** | **-** |
| VLDL-5 | **√** | **√** | **√** | **√** | **-** | **-** | **-** |
| VLDL-6 | **√** | **√** | **√** | **√** | **-** | **-** | **-** |
| IDL | **√** | **√** | **√** | **√** | **-** | **-** | **√** |
| All LDL | **√** | **√** | **√** | **√** | **-** | **-** | **√** |
| LDL-1 | **√** | **√** | **√** | **√** | **-** | **-** | **√** |
| LDL-2 | **√** | **√** | **√** | **√** | **-** | **-** | **√** |
| LDL-3 | **√** | **√** | **√** | **√** | **-** | **-** | **√** |
| LDL-4 | **√** | **√** | **√** | **√** | **-** | **-** | **√** |
| LDL-5 | **√** | **√** | **√** | **√** | **-** | **-** | **√** |
| LDL-6 | **√** | **√** | **√** | **√** | **-** | **-** | **√** |
| All HDL | **√** | **√** | **√** | **√** | **√** | **√** | **-** |
| HDL-1 | **√** | **√** | **√** | **√** | **√** | **√** | **-** |
| HDL-2 | **√** | **√** | **√** | **√** | **√** | **√** | **-** |
| HDL-3 | **√** | **√** | **√** | **√** | **√** | **√** | **-** |
| HDL-4 | **√** | **√** | **√** | **√** | **√** | **√** | **-** |

Supplementary Table 1. An overview of the parameters obtained using the ^1^H-NMR lipoprotein subclassification method.

| **Lipid Sub-Fraction** | **Concentration (mg/dL)** | |
| --- | --- | --- |
|  | ***Patient*** | ***Control*** |
| **Total Plasma** |  |  |
| Cholesterol | 214.60 (± 51.1) | 216.88 (± 50.9) |
| Free Cholesterol | 67.70 (± 14.3) | 68.4 (± 14.20) |
| Triglycerides | 105.7 (± 68.81) | 106.18 (± 70.0) |
| LDL-Cholesterol | 114.9 (± 34.69) | 116.28 (± 34.90) |
| HDL-Cholesterol | 67.03 (± 16.24) | 67.61 (± 16.41) |

Supplementary Table 2. Concentrations of total plasma cholesterol, free cholesterol and triglycerides in MS patients and healthy controls. Concentrations are quoted in mg/dL ± standard deviation.

|  | Model R^2^ | Model  P-Value | V1PL | V2TG | V2PL |
| --- | --- | --- | --- | --- | --- |
| LOO1 | 0.48 | 8.47907E-06 | 0.014 | 0.004 | 0.014 |
| LOO2 | 0.48 | 7.69336E-06 | 0.009 | 0.003 | 0.014 |
| LOO3 | 0.51 | 2.08679E-06 | 0.006 | 0.001 | 0.006 |
| LOO4 | 0.49 | 5.44127E-06 | 0.013 | 0.005 | 0.020 |
| LOO5 | 0.47 | 8.69054E-06 | 0.010 | 0.004 | 0.014 |
| LOO6 | 0.51 | 2.20107E-06 | 0.011 | 0.003 | 0.010 |
| LOO7 | 0.48 | 7.42451E-06 | 0.009 | 0.003 | 0.014 |
| LOO8 | 0.49 | 4.09666E-06 | 0.008 | 0.003 | 0.011 |
| LOO9 | 0.49 | 4.46746E-06 | 0.004 | 0.002 | 0.007 |
| LOO10 | 0.48 | 6.5764E-06 | 0.012 | 0.004 | 0.015 |
| LOO11 | 0.49 | 4.2431E-06 | 0.007 | 0.002 | 0.009 |
| LOO12 | 0.48 | 6.41244E-06 | 0.010 | 0.004 | 0.014 |
| LOO13 | 0.46 | 1.3048E-05 | 0.010 | 0.003 | 0.013 |
| LOO14 | 0.48 | 6.2049E-06 | 0.009 | 0.003 | 0.014 |
| LOO15 | 0.48 | 7.53966E-06 | 0.010 | 0.004 | 0.015 |
| LOO16 | 0.48 | 6.86041E-06 | 0.014 | 0.006 | 0.022 |
| LOO17 | 0.48 | 7.5189E-06 | 0.009 | 0.005 | 0.019 |
| LOO18 | 0.51 | 2.31859E-06 | 0.005 | 0.001 | 0.005 |
| LOO19 | 0.52 | 1.16125E-06 | 0.005 | 0.003 | 0.014 |
| LOO20 | 0.48 | 6.71033E-06 | 0.008 | 0.003 | 0.014 |
| LOO21 | 0.50 | 3.36502E-06 | 0.002 | 0.002 | 0.010 |
| LOO22 | 0.48 | 5.8468E-06 | 0.010 | 0.003 | 0.011 |
| LOO23 | 0.48 | 7.53797E-06 | 0.010 | 0.004 | 0.015 |
| LOO24 | 0.47 | 9.38576E-06 | 0.029 | 0.008 | 0.024 |
| LOO25 | 0.46 | 1.58951E-05 | 0.016 | 0.004 | 0.014 |
| LOO26 | 0.48 | 6.45236E-06 | 0.010 | 0.004 | 0.015 |
| LOO27 | 0.48 | 8.4988E-06 | 0.013 | 0.006 | 0.020 |

Supplementary Table 3. Results of leave-one-out cross validation for optimal regression model. Statistical measures of fit for each leave-one-out model displayed as Model R^2^ value. Corresponding p-value for the model and for each VLDL predictor coefficient provided. Total of 27 leave-one-out cross validations performed.
